# How Can Plant DNA Viruses Evade siRNA-Directed DNA Methylation and Silencing?

**DOI:** 10.3390/ijms140815233

**Published:** 2013-07-24

**Authors:** Mikhail M. Pooggin

**Affiliations:** University of Basel, Department of Environmental Sciences, Botany, Schönbeinstrasse 6, Basel 4056, Switzerland; E-Mail: mikhail.pooggin@unibas.ch; Tel.: +41-61-267-2977; Fax: +41-61-267-3504

**Keywords:** plant virus, DNA virus, geminivirus, pararetrovirus, silencing, siRNA, RNA-directed DNA methylation, cytosine methylation, silencing evasion, suppressor protein

## Abstract

Plants infected with DNA viruses produce massive quantities of virus-derived, 24-nucleotide short interfering RNAs (siRNAs), which can potentially direct viral DNA methylation and transcriptional silencing. However, growing evidence indicates that the circular double-stranded DNA accumulating in the nucleus for Pol II-mediated transcription of viral genes is not methylated. Hence, DNA viruses most likely evade or suppress RNA-directed DNA methylation. This review describes the specialized mechanisms of replication and silencing evasion evolved by geminiviruses and pararetoviruses, which rescue viral DNA from repressive methylation and interfere with transcriptional and post-transcriptional silencing of viral genes.

## 1. Introduction

DNA viruses accumulate in the nuclei of infected plant cells as multiple circular minichromosomes. which resemble the host plant chromosomes in that the viral DNA is packaged into nucleosomes forming chromatin. Furthermore, viral minichromosomes are transcribed by the host Polymerase II (Pol II), which generates capped and polyadenylated viral RNAs, similar to mRNAs generated by Pol II from most plant protein-coding genes. Thus, viral minichromosomes must encounter the nuclear pathways that regulate host gene expression and chromatin states. However, DNA viruses have evolved specialized mechanisms of replication that differ from those replicating the plant chromosomes. These replication mechanisms can potentially rescue viral minichromosomes from repressive chromatin marks that silence certain plant genes and repetitive DNA elements in transcriptionally-inactive heterochromatic regions. Some of the repressive chromatin marks are established by the RNA-directed DNA methylation (RdDM) pathway. RdDM is a nuclear branch of the plant RNA silencing machinery that regulates gene expression and defends against invasive nucleic acids such as transposons, transgenes and viruses. The plant RNA silencing machinery generates 21, 22 and 24 nt small RNAs which are broadly classified into miRNAs and short interfering RNAs (siRNAs). These small RNAs serve as guide molecules for the silencing complexes that repress genes post-transcriptionally and/or transcriptionally in a sequence-specific manner. The transcriptional silencing through *de novo* DNA methylation is directed by 24-nt siRNAs, the most diverse and abundant class of plant small RNAs. Likewise, plant DNA viruses spawn massive quantities of viral 24-nt siRNAs which can potentially silence viral DNA. In this review, I will focus mainly on the nuclear events in life cycles of plant DNA viruses and describe the strategies of silencing evasion evolved by *Geminiviridae* (geminiviruses) and *Caulimoviridae* (pararetroviruses), the two major families of plant DNA viruses. The third DNA virus family, *Nanoviridae*, is discussed, because little is known about interactions of nanoviruses with the plant silencing system. Since they resemble geminiviruses in DNA replication mechanisms [1], the findings for geminiviruses could be extrapolated to nanoviruses. The post-transcriptional RNA silencing mechanisms which contribute to plant defenses against both RNA and DNA viruses, and the biogenesis and function of the three major classes viral siRNAs including 21-nt and 22-nt classes have been reviewed comprehensively [2–5]. Various silencing suppressor proteins encoded by plant viruses have also been reviewed [6,7], and I will focus only on those encoded by DNA viruses and describe emerging evidence that viral suppressor proteins may have effector functions in suppressing plant innate immunity [8].

## 2. Plant DNA Methylation

DNA methylation at cytosine nucleotides (5meC) is a reversible epigenetic mark that plays a key role in regulation of gene expression and chromatin states in most eukaryotes. Plants and mammals require cytosine methylation for proper development and genome defense against transposons [9,10]. In mammals, methylation occurs predominantly at symmetric CG sites and, following DNA replication, can be maintained by DNA METHYLTRASFERASE 1 (DNMT1). DNMT1 recognizes hemimethylated double-stranded DNA (dsDNA) with the help of methyl binding domain proteins and catalyzes methylation of symmetric cytosines on the newly-synthesized strand. Establishment of cytosine methylation on unmethylated dsDNA is catalyzed by *de novo* methyltransferases DNMT3a and DNMT3b. Furthermore, methylated dsDNA can be actively demethylated, which ensures dynamic regulation of chromatin states during development and in response to environmental cues. Generally, methylated DNA is repressed transcriptionally, because it is packed into heterochromatin inaccessible to RNA polymerases, whereas unmethylated DNA is present in open actively-transcribed euchromatin.

**Figure 1:**
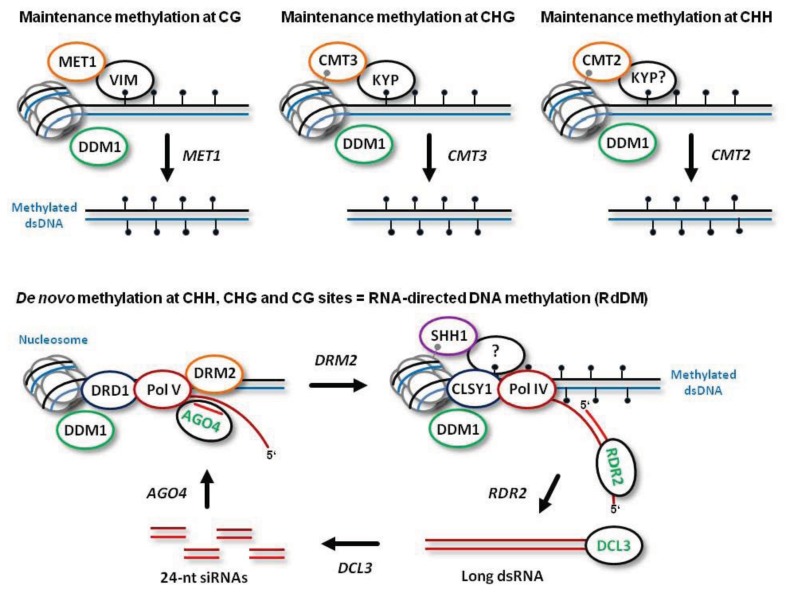
Models for maintenance methylation and RdDM at the plant genome loci. (Based mostly on the findings using the model plant *Arabidopsis*). The plant dsDNA associated with nucleosomes is depicted as solid lines and the methylated cytosines at one or both strands indicated with black lollypops. Following DNA replication, cytosine methylation at CG, CHG and CHH sites of the newly-synthesized strand (blue) is catalyzed by the maintenance methyltransferates MET1, CMT3, and CMT2, respectively, with the help of co-factors VIM and KYP that recognize hemimethylated dsDNA; CMT3 and CMT2 also bind the repressive histone methylation mark H3K9me2 indicated as grey lollypops. The RNA-directed DNA methylation (RdDM) pathway establishing methylation of dsDNA *de novo* is catalyzed by DRM2 that interacts with the DRD1-Pol V complex generating a scaffold transcript. The nascent scaffold transcript is targeted by the 24-nt siRNA-AGO4 complex. Following DRM2-catalyzed *de novo* methylation of both DNA strands, Pol IV with the help of SHH1 (binding H3K9me2) and CLSY1 initiates siRNA biogenesis. The Pol IV transcript is converted by RDR2 to dsRNA. The resulting dsRNA is processed by DCL3 into 24-nt siRNA duplexes. The duplexes are handed over to AGO4 to form the silencing complexes with a single-stranded siRNA guide. This completes an siRNA amplification loop that reinforces RdDM-mediated transcriptional silencing. The chromatin remodeler DDM1 facilitates the access of all the methyltransferases to dsDNA.

In flowering plants, cytosines in all possible sequence contexts can be methylated, including symmetric (CG and CHG, where H is A, C, or T) and asymmetric (CHH). *De novo* establishment of methylation at CG, CHG and CHH sites is catalyzed by DOMAINS REARRANGED METHYLTRANSFERASE 2 (DRM2), the plant homolog of mammalian DNMT3a and DNMT3b, which requires 24-nt siRNA guide molecules and other components of the RdDM pathway (Figure 1; see below for more details). Following DNA replication, symmetric CG methylation is maintained by DNA METHYLTRANSFERASE 1 (MET1), the plant homolog of mammalian DNMT1, which recognizes hemimethylated dsDNA with the help of CG-specific methyl binding proteins VARIANT IN METHYLATION 1 (VIM1), VIM2 and VIM3 [11] (Figure 1). Symmetric CHG methylation is maintained by CHROMOMETHYLASE 3 (CMT3), a plant-specific methyltransferase that recognizes dimethylated histone 3 tails at lysine 9 (H3K9m2) on the nucleosomes (Figure 1). In this process, CHG methylation at the template strand is recognized by the H3K9m2 methyltransferase KRYPTONITE (KYP), which can bind methylated cytosines in both CHG and CHH context [12]. Thus, CHG methylation is maintained through a reinforcing loop of DNA and histone (H3K9) methylation. Recently, a homolog of CMT3, CMT2, has been implicated in maintenance methylation at CHH sites [13]. Like CMT3, CMT2 is recruited through direct recognition of the methylated histone H3K9me2 and does not require siRNA guides or other components of RdDM (previously thought to be the only pathway maintaining CHH methylation). Furthermore, indirect recognition of the hemimethylated DNA by CMT2 may also require KYP that binds methylated CHH sites (Figure 1).

Both maintenance methylation and RdDM are facilitated by a chromatin remodeler DEFFICIENT IN DNA METYLATION 1 (DDM1). Indeed, 70% of CG, CHG and CHH methylation is lost in *ddm1* mutant plants. It is believed that maintenance methylation does not take place on naked dsDNA immediately following passage of the DNA replication fork, and that cytosine methylation occurs in a nucleosomal context involving both core and linker histones [14]. DDM1 remodels heterochromatin by removing the repressive linker histone H1 [13]. Obviously, all the DNA methyltransferases need the access to DNA, which can be facilitated by DDM1 (Figure 1). Together, DDM1 and RdDM synergize to maintain all the cytosine methylation in the plant genome [13].

Other factors required for normal DNA methylation include those that have direct or indirect impact on the levels of *S*-adenosyl-l-methionine (SAM), the donor of methyl groups.

## 3. Mechanism of RNA-Directed DNA Methylation (RdDM)

RdDM is mediated by two plant-specific DNA-dependent RNA polymerases, Pol IV and Pol V: Pol IV functions to initiate siRNA biogenesis, while Pol V generates scaffold transcripts that recruit downstream RdDM factors [15]. Both Pol IV and Pol V are plant-specific enzymes that have evolved from Pol II and share several core Pol II subunits. However, little is known about promoters and other regulatory elements driving transcription at the RdDM loci; the transcripts generated by Pol V and Pol IV were not precisely mapped.

The model depicted in Figure 1 (based mostly on the findings using the model plant *Arabidopsis*) states that Pol V scaffold transcripts are produced at DNA loci to be methylated *de novo*. The nascent scaffold transcript is targeted by an ARGONAUTE 4 (AGO4) protein complex containing a 24-nt siRNA guide molecule via complementary interaction of the siRNA and the scaffold RNA. AGO4 belongs to a family comprising ten members, most of which possess catalytic activity required for sequence-specific cleavage of their target RNAs and subsequent gene silencing at both transcriptional and post-transcriptional levels [16,17]. Besides catalyzing cleavage of the nascent Pol V transcript, AGO4 interacts with Pol V itself. Together, these interactions are required for recruitment of the methyltransferase DRM2 (or its homolog DRM1) and for subsequent *de novo* methylation of both DNA strands (Figure 1). Other factors that facilitate Pol V transcription and DRM2 recruitment include DEFECTIVE IN RNA-DIRECTED DNA METHYLATION 1 (DRD1), DEFECTIVE IN MERISTEM SILENCING 3 (DMS3) and RNA-DIRECTED DNA METHYLATION 1 (RDM1). These proteins form a complex proposed to unwind dsDNA in front of Pol V (via a putative DNA translocase/ATPase activity of DRD1) and to mediate recruitment of DRM2 to the AGO4-bound scaffold transcript. Following AGO4-catalyzed cleavage of the scaffold transcript, the released siRNA-AGO4 complex may bind the complementary DNA and thereby define the region to be methylated by DRM2 [15]. Other members of the nuclear AGO clade, AGO6 and AGO9, which display tissue specific expression, might also function in RdDM together with, or in place of AGO4 [18].

The biogenesis of 24-nt siRNAs at the RdDM loci is initiated by Pol IV transcription. Pol IV transcripts are then converted to double-stranded RNA (dsRNA) by RNA-DEPENDENT RNA-POLYMERASE 2 (RDR2) (Figure 1). RDR2 belongs to a family with at least three functional enzymes involved in the biogenesis of distinct classes of endogenous plant siRNAs and viral secondary siRNAs. Thus, RDR6 generates dsRNA precursors of plant trans-acting siRNAs (tasiRNAs), which have been precisely mapped [19,20], while RDR1 and RDR6 together are involved in the biogenesis of secondary siRNAs derived from RNA viruses [21–23]. RDR2-dependent dsRNA precursors of 24-nt siRNAs have not been mapped, and it is presumed that RDR2 converts to dsRNA a complete Pol IV transcript, or generates Okazaki-like fragments on the nascent Pol IV transcript [15]. Notably, RDR2 and Pol IV form a complex, and RDR2 has no activity in the absence of Pol IV [24]. Together, Pol IV and RDR2 are required for the biogenesis of virtually all endogenous plant 24-nt siRNAs.

Pol IV is localized at the target loci through interaction with SAWADEE HOMEODOMAIN HOMOLOG 1 (SHH1) that recognizes H3K9me2 [25]. Furthermore, Pol IV occupancy at actively-transcribed, siRNA-generating loci may also require methyl binding protein activity, because Pol IV is believed to transcribe methylated DNA following *de novo* methylation (Figure 1). Pol IV transcription of methylated DNA at the RdDM loci would amplify 24-nt siRNAs to reinforce silencing *in cis*, maintain methylation following replication, and enable *de novo* methylation of homologous DNA loci *in trans*.

*De novo* methylation might also occur at some Pol II loci via targeting of nascent Pol II transcripts by 24-nt siRNAs [26–28]. In fact, such events might trigger *de novo* methylation and transcriptional silencing of active long-terminal repeat (LTR) retrotransposons (whose genomic RNA is generated by Pol II) following their transposition at new loci.

RdDM and Pol IV activity require CLASSY 1 (CLSY1), a putative ATP-dependent nucleic acid translocase predicted to evict nucleosomes and unwind dsDNA (Figure 1). As discussed above, the chromatin remodeler DDM1 might also facilitate RdDM by removing the repressive histone H1. Establishment of other repressive histone modifications at RdDM loci is catalyzed by a Jumonji domain protein JMJ14 and HISTONE DEACETYLASE 6 (HDA6). JMJ14 demethylates histone H3 lysine 4, thus removing the mark associated with active chromatin. Likewise, HDA6 removes acetyl groups from histone lysines (*i.e.*, active chromatin marks), which is a prerequisite for their subsequent methylation creating the repressive marks such as H3K9me2 [15].

The final step in the biogenesis of endogenous 24-nt siRNAs is accomplished by DICER-LIKE 3 (DCL3), an RNase III-like enzyme that belongs to a family of four prototype members [29]. DCL3 catalyzes processing of RDR2-dependent dsRNA into 24-nt siRNA duplexes (Figure 1). These duplexes are then methylated at the 3′-terminal nucleotides’ hydroxyls by HUA ENHANCER 1 (HEN1) and sorted by AGO4, AGO6, or AGO9 to form the silencing complexes containing a single-stranded 24-nt siRNA guide molecule [18]. Either strand of the siRNA duplex can get incorporated into the AGO complex, which enables targeting of both sense and antisense transcripts, potentially generated at the RdDM loci.

## 4. DNA Demethylation

DNA demethylation can occur passively through several rounds of DNA replication in the absence of efficient maintenance methylation, or actively through enzymatic activities. In plants, DNA glycosylases have been implicated in active removal of 5meC from DNA [9,30]. These include DEMETER (DME) which controls imprinting in reproductive tissues, REPRESSOR OF SILENCING 1 (ROS1) initially identified as suppressor of transcriptional silencing of a plant promoter-driven transgene, and two DEMETER-LIKE enzymes (DML2 and DML3) which, together with ROS1, counteract excessive methylation at several hundred loci across the genome [31–33]. The DNA glycosylases can remove repressive cytosine methylation marks in all the sequence contexts without the need for DNA replication and thereby release transcriptional silencing. However, it is not clear what provides sequence specificity for these enzymes. Animals apparently lack 5meC DNA glycosylases and demethylation involves excision of de-aminated and/or oxidized derivatives of 5meC [34].

A crosstalk between demethylation and *de novo* methylation pathways has been recently illustrated by the finding that ROS1 expression is controlled by the RdDM pathway and mutations in Pol IV and Pol V cause transcriptional silencing at the ROS1 target loci [35].

## 5. Replication Modes of Geminiviruses

**Figure 2:**
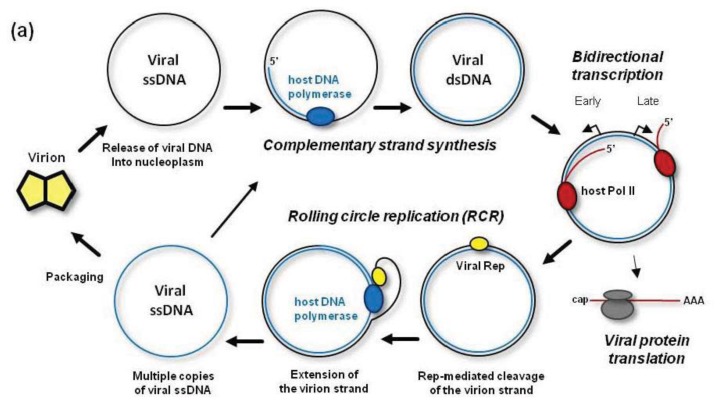

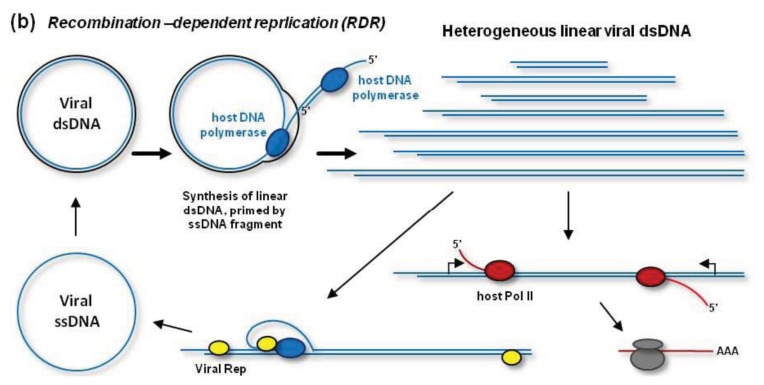
Models for RCR and RDR modes of geminivirus DNA replication. (**a**) RCR. The viral circular ssDNA is released from the virion (yellow) into the nucleus. The host DNA polymerase synthesizes the complementary strand, yielding circular covalently-closed dsDNA. This dsDNA serves as a template for bidirectional transcription of the early leftward (Rep) and the late rightward (coat protein) genes. Viral mRNAs are transported to the cytoplasm. Following translation, Rep moves to the nucleus to initiate replication of the viral dsDNA by a rolling circle replication (RCR) mechanism. Rep (in yellow) nicks the virion strand in the origin of replication and recruits the host DNA polymerase to extend 3′-end of the cleaved virion strand on the complementary strand template. As the extension progresses, the polymerase complex, associated with Rep covalently linked to the 5′-end of the virion strand, displaces the virion strand. After one or more rounds of replication on the circular complementary strand template, Rep nicks and religates the displaced virion strand extended by one or more copies of the newly-synthesized virion strand and thereby releases one or more copies of circular ssDNA. The resulting circles can re-enter the replication cycle or get packaged into virions; (**b**) The circular covalently-closed dsDNA is invaded by a short viral DNA primer. The primer is extended by the host DNA polymerase on the circular viral template strand. After (or during) one or more rounds of replication, the newly-synthesized linear ssDNA gets fully or partially converted to linear dsDNA by the same (or another) DNA polymerase complex. Thus, RDR generates a heterogeneous population of linear dsDNAs. The long linear dsDNAs that harbor two or more origins of replication are transcribed by Pol II in both orientations to generate viral mRNAs. Following translation, Rep initiates replication of the long linear dsDNA with two or more origins of replication. The replicational release of ssDNA from the multimeric linear dsDNA generates circular ssDNA that can re-enter the replication cycle or get packaged.

The family *Geminiviridae* comprises circular single-stranded DNA (ssDNA) viruses with 2.5–3.2 kb genomes [36]. The *Begomovirus* genus contains monopartite or bipartite geminiviruses with an additional circular ssDNA component of similar size (DNA-B). Viral ssDNA is encapsidated by viral coat protein in twinned (geminate) virions. The life cycle of geminiviruses, their replication and gene expression strategies have been comprehensively reviewed [37,38]. According to the current model (Figure 2), following insect injection into a plant cell, the viral particle is targeted via a coat protein-based nuclear localization signal to the nucleus, where viral ssDNA is released into nucleoplasm. The circular ssDNA is then converted to circular dsDNA by the host DNA polymerase and other components of the DNA repair machinery. In genus *Begomovirus*, the complementary strand synthesis is primed by an RNA primer [39]. By contrast, in genus *Mastrevirus*, a nested set of complementary strand DNA primers with major species ranging from 78 to 88 nts were found to be associated with virion-derived ssDNA [40].

Following complementary strand synthesis, the resulting covalently-closed circular dsDNA gets associated with nucleosomes [41,42] and transcribed by the host Pol II (Figure 2A). Pol II transcribes the viral minichromosome in the leftward orientation to generate mRNAs for viral replication-initiator protein (Rep) and other proteins assisting replication and transcription. At a later stage, the minichromosome is transcribed by Pol II in the rightward orientation to generate mRNA for coat protein [37,43]. In begomovirus-infected plants, the number of nucleosomes per viral minichromosome is varying between 11 and 12, presumably representing transcriptionally active states, and 13, representing inactive state [42].

After production by the cytoplasmic ribosomes, the viral Rep protein moves to the nucleus to initiate rolling circle replication (RCR) of the viral dsDNA that had given rise to the Rep mRNA. Rep is the only viral protein essential for the RCR mechanism generating multiple copies of circular ssDNA. Rep initiates RCR by nicking the virion strand of dsDNA in a conserved nonanucleotide sequence of the replication origin and by recruiting the host DNA polymerase complex. The polymerase uses the circular complementary strand as a template to extend 3′-end of the cleaved virion strand. During this process, the virion strand with Rep covalently linked to its 5′-end is displaced from the template strand (Figure 2A). Rep helicase activity has also been implicated in a post-initiation phase of RCR [44]. After one or more rounds of RCR, Rep (being associated with the polymerase complex) nicks and ligates the displaced virion strand extended with one or more copies of the newly-synthesized virion strand, and thereby releases circular ssDNA from the complex. Thus, multiple circles of viral ssDNA are synthesized on one complementary ssDNA circle (Figure 2A). These circles can re-enter the replication cycle, or get packaged into virions at later stages of infection, when viral coat protein is accumulated. As a result of RCR, multiple copies of viral minichromosomes accumulate in the initially-infected nucleus and eventually in the nuclei of other cells that are infected by cell-to-cell and long-distance movement of viral particles.

In addition to RCR, geminiviruses can replicate their dsDNA by a recombination-dependent replication (RDR) mechanism [38,45–47]. According to a model shown in Figure 2B, RDR is initiated by a viral ssDNA fragment that invades a homologous region of the circular dsDNA with the help of the host recombination enzymes. Then the host DNA polymerase extends the invaded ssDNA on a template strand. During (or after) one or more rounds of the extension on the circular template, the resulting linear ssDNA is converted to dsDNA by the DNA polymerase complex primed by a short complementary fragment of viral DNA (or RNA). Thus, RDR generates a heterogeneous population of linear dsDNAs, which accumulate at high levels during viral infection and become targeted for cytosine methylation [48] (see below). RDR priming does not require Rep activity [38]. However, Rep may release circular ssDNA from the heterogeneous linear dsDNA, which contains two or more origins of replication [49] (Figure 2B). In fact, such mechanism is responsible for the release of circular ssDNA from partial dimer clones of geminiviruses widely used for experimental inoculations.

The efficient mechanism of RDR evolved by geminiviruses explains why recombination is a major driving force for their evolution and a frequent cause of epidemics. Indeed, if two geminiviruses enter the same nucleus, RDR will very likely produce a wide variety of chimeric genomes. It should be stressed, however, that Rep-mediated RCR is essential for systemic infection of the plant and for formation of the virions with circular ssDNA, which are transmitted by insects from plant to plant. Thus, both modes of replication are required for robust infection and spread of geminiviruses. The following section describes how RCR and RDR help geminiviruses evade repressive cytosine methylation and transcriptional silencing.

## 6. Evasion of Maintenance Methylation and RdDM by Geminiviruses

It has been proposed that cytosine methylation is one of the major host defense mechanisms against geminiviruses and therefore these viruses have evolved different suppressor proteins to interfere with repressive methylation and transcriptional silencing of viral DNA [50]. Here I argue that geminiviruses can evade repressive methylation simply via efficient Rep-dependent replication as has been suggested earlier [48,51].

Experimental evidence based on bisulfite treatment of total DNA from geminivirus-infected plants, followed by PCR amplification and sequencing of the virion strand, shows that 50% to 99% (depending on the virus or the host used) of viral DNA is not methylated [52,53]. Note that technical biases of the bisulfite sequencing method may have prevented correct evaluation of the percentage of 5meC in viral DNA, as discussed by Paprotka *et al.* [48]. Interestingly, methylated cytosines were not randomly distributed between the viral molecules, but concentrated in a small fraction of densely methylated molecules [53]. Hence, a large fraction of viral DNA is not methylated at all. Since the most abundant form of viral DNA is circular ssDNA that gets encapsidated into virions, the above findings imply that this form is not methylated and therefore maintenance methylation does not occur during Rep-mediated RCR. As discussed above, maintenance methylation likely occurs in a nucleosomal context (Figure 1). During the first round of RCR the nucleosomes are removed from the replicating viral DNA and their formation is prevented by continuous rounds of replication displacing newly-synthesized ssDNA (Figure 2A). Moreover, the latter ssDNA is only transiently associated with the template strand, thus preventing an access of the hemimethylated dsDNA-binding proteins required for recruitment of methyltransferases (Figure 1). Likewise, the RDR mechanism generating heterogeneous linear dsDNA on a circular dsDNA template (Figure 2B) is not compatible with maintenance methylation. The variable levels of cytosine methylation detected by bisulfite sequencing in all the sequence contexts [52,53] likely reflect the amounts of *de novo* methylated viral dsDNA in circular or linear forms, both containing the virion strand. Using the bisulfite sequencing approach to evaluate a methylation status of the complementary strand (*i.e.*, RCR template) revealed 36% to 45% of methylation in all the sequence contexts [51]. Hence, a large fraction of viral dsDNA is also not methylated. Taking the above findings and considerations together, detectable methylation of geminiviral DNA is established *de novo*, possibly through RdDM using viral 24-nt siRNA guides. Potential targets of RdDM could be both circular dsDNA and heterogeneous linear dsDNA, which undergo transcription (Figure 2), because RdDM requires on-going transcription at the endogenous target loci (Figure 1).

The lack of maintenance methylation during geminivirus replication is further supported by the findings that geminiviral clones methylated *in vitro* gave rise to unmethylated dsDNA progeny in plant protoplasts, although viral DNA replication was inhibited compared to unmethylated controls [54,55]. These findings illustrate the repressive nature of cytosine methylation, likely inhibiting initial transcription of viral genes. However, more importantly, they demonstrate the ability of geminiviruses to resurrect viral DNA from repressive methylation by evading maintenance methylation during replication.

The methylation status of viral dsDNA in the above-described protoplast studies was evaluated by treatment of total DNA with methylation sensitive enzymes, followed by Southern blot hybridization with virus-specific probes. This approach did not reveal any substantial methylation of viral circular dsDNA in plants infected with different geminiviruses [48,54,56]. The conflicting results obtained with two different methods can be explained by the inability of PCR-based bisulfite sequencing to discriminate between different forms of viral DNA. To resolve this problem, more advanced methods have been applied, using treatment of total DNA with methylation-dependent enzyme McrBC, followed by 1-D or 2-D gel separation and Southern blot analysis or detection with 5meC-specific antibodies [48]. This study has confirmed that circular dsDNA, the template for both replication and transcription, is not methylated. The only viral DNA form that possessed detectable cytosine methylation is heterogeneous linear dsDNA, the product of RDR. Therefore, the extremely variable levels of DNA methylation detected by bisulfite sequencing, ranging for wild-type geminiviruses from 1.25% to 50%–60% [52,53], may reflect the amounts of heterogeneous linear dsDNA accumulated in the respective virus-host systems. The highest methylation level (88%) was reported for an intergenic region of the curtovirus *Beet curly top virus* (BCTV) mutant lacking an L2 gene [52]. *Arabidopsis* plants recover from this mutant virus infection and accumulate very low levels of highly methylated viral DNA. The residual replication of this defective virus in recovered tissues may proceed mainly by RDR that generates linear dsDNA, the target for methylation. Another explanation is that the BCTV L2 protein acts an active suppressor of cytosine methylation [57] (discussed below).

It has been reported that plants deficient in cytosine methylation exhibit increased sensitivity to geminivirus infection [52]. Thus, enhanced disease symptoms were observed for the begomovirus *Cabbage leaf curl virus* (CaLCuV) and the curtovirus BCTV in *Arabidopsis* mutants lacking core components of maintenance methylation or RdDM, which included DRM1/2, Pol IV/V, DDM1, MET1, CMT3, KYP, DCL3, or AGO4. However, mutant plants lacking RDR2, which is also required for RdDM (Figure 1B), did not display enhanced symptoms. Moreover, the mutant plants displaying enhanced symptoms accumulated the wild-type levels of viral DNA [52]. Hence, viral DNA replication is not “de-repressed” in the absence of core components of RdDM or maintenance methylation pathways and, in wild type plants, repressive cytosine methylation can be effectively evaded, likely by Rep-mediated replication of viral DNA. Consistent with this notion, another study did not reveal increased titres or enhanced symptoms of CaLCuV in *Arabidopsis* mutants lacking Pol IV, RDR2, DCL3, or AGO4 [58]. Notably, viral 24-nt siRNAs were normally produced in all these mutants, except *dcl3*, indicating that the biogenesis of viral 24-nt siRNAs does not require the RdDM components essential for production of dsRNA precursors of endogenous 24-nt siRNAs (see below).

## 7. Suppression of Cytosine Methylation and Transcriptional Silencing by Geminiviral Proteins

Geminiviral proteins implicated in suppression of cytosine methylation and transcriptional silencing include AC2/AL2/C2/L2 homologs encoded by *Begomovirus* and *Curtovirus* genera and betaC1 encoded by betasatellites associated with some begomoviruses. Since these proteins are not conserved in all genera of *Geminiviridae*, other viral protein(s) may suppress transcriptional silencing (see below) or, as argued here, all geminiviruses should be able to evade cytosine methylation through Rep-dependent replication.

The begomovirus AC2/AL2/C2 gene encodes a transcriptional activator (TrAP) required for activation of late viral genes in the nucleus [37,43]. This protein was also shown to suppress post-transcriptional silencing and its nuclear localization was required for this activity [59,60]. Notably, the suppressor activity of AC2 from two Old World begomoviruses correlated with upregulation of a common subset of host genes including WERNER-LIKE EXONUCLEASE 1 (WEL1), which may act as negative regulators of RNA silencing [60]. Thus, AC2 appears to suppress post-transcriptional silencing indirectly via transcriptional activation of the host genes. Interestingly, the *WEL1* gene that codes for a putative silencing suppressor [60] seats in a transcriptionally-silent locus containing seven *WEL1* paralogs in head-to-tail orientation [60]. Transcriptional silencing of this repetitive DNA locus, likely associated with repressive chromatin marks, might be reversed by the viral TrAP activity. It should be mentioned that in addition to its antisilencing function, *WEL1* may also function in viral DNA replication, because it encodes a putative 3′–5′ exonuclease [60] which resembles the WERNER exonuclease involved in DNA replication, recombination and repair.

It has been demonstrated that the begomovirus CaLCuV AL2 and the curtovirus BCTV L2 can reverse transcriptional silencing at transgenic and some endogenous loci repressed by cytosine methylation [57]. The reversal of silencing correlated with partial reduction of non-CG methylation at the respective loci as well as with genome-wide reduction in CHG methylation. In this process, CaLCuV AL2 did not require the C-terminal transcriptional activation domain [57]. This is in contrast to a homologous AC2 protein from the Old Word begomovirus *Mungbean yellow mosaic virus*, which needs this domain to activate the host genes and suppress post-transcriptional silencing [60]. Surprisingly, both AL2 from the New World begomoviruses (CaLCuV and TGMV) and L2 from the curtovirus BCTV reverse transcriptional silencing and cytosine methylation by a mechanism that does not require their nuclear localization. These proteins interact with and inactivate ADENOSINE KINASE (ADK), a cytoplasmic enzyme involved in the methyl cycle producing SAM, the donor of methyl groups [50]. Curiously, AL2/L2-mediated inactivation of ADK was required for suppression of both transcriptional [57] and post-transcriptional [61] silencing.

A different mechanism of silencing suppression was reported for C2 of the curtovirus *Beet severe curly top virus*. This protein interacts with SAM DECARBOXYLASE 1 and thereby interferes with the methyl cycle and DNA methylation [62]. Given that different mechanisms were reported for the closely homologous proteins such as C2 and L2 from curtoviruses as well as AC2 and AL2 from begomoviruses, further research should clarify their activities in the nucleus and the cytoplasm.

Some monopartite begomoviruses are associated with betasatellites that enhance disease symptoms [63]. Betasatellites code for a single protein, betaC1, reported to act as a suppressor of post-transcriptional silencing [64,65]. Like begomoviral AC2/C2, betaC1 from a betasatellite of *Tomato yellow leaf curl China virus* (TYLCCNV) is a nuclear protein and its nuclear localization is required for silencing suppression [64]. The same betaC1 protein was also reported to reverse transcriptional silencing and CHG methylation through inactivation of S-ADENOSYL HOMOCYSTEINE HYDROLASE (SAHH), a methyl cycle enzyme required for synthesis of the methyl donor SAM [53]. Curiously, betaC1 requires an intact nuclear localization signal for the cytoplasmic interaction with SAHH. In the presence of betasatellite, cytosine methylation of the TYLCCNV virion strand was reduced from 5.4% to 1.25%. Since TYLCCNV infection or expression of the TYLCCNV C2 protein failed to reverse transcriptional silencing or cytosine methylation at endogenous loci, betaC1 was proposed to functionally substitute for a loss-of-function mutation in the TYLCCNV C2 gene [53]. However, very low methylation of TYLCCNV DNA in the absence of betasatellite (5.4%) would argue against the absolute necessity for a geminivirus to possess a suppressor of cytosine methylation.

Enhanced symptoms of geminiviral disease in the presence of betasatellite as well as in the methylation-deficient mutant plants described above could be explained by possible involvement of hypomethylation of the host genome in anti-viral defense responses. The symptom severity could be proportional to the expression levels of host defense genes which are induced though demethylation in response to viral infection. The activities of certain geminiviral viral proteins might be recognized by the immune receptors from NUCLEOTIDE BINDING-LEUCINE RICH REPEAT (NB-LRR) family, which induce expression of defense genes in response to both non-viral and viral pathogens [8]. It remains to be investigated if the immune responses to viral infection require active demethylation of the host genome, triggered by recognition of viral proteins.

It has been reported that begomoviral Rep has the ability to reverse transcriptional silencing and reduce CG methylation at endogenous loci, possibly through Rep-mediated downregulation of *MET1* [66]. However, in addition to *MET1*, the transcript levels of *CMT3* and *ROS1* (but not *DRM2*) were also downregulated by transient expression of Rep or by geminivirus infection. It is unclear how the downregulation of the maintenance methyltransferases and the demethylase together would reduce CG methylation and reverse transcription silencing at the endogenous loci, and whether these effects of Rep are important for viral infection. The ability of geminiviral Rep to modify cell cycle and trigger host DNA reduplication [37,67,68] may explain the reduced levels of cytosine methylation at endogenous loci in the Rep transgenic plants upon induction of Rep expression [66]. As described above, Rep-mediated replication of TYLCCNV failed to suppress transcriptional silencing or cytosine methylation [53]. Moreover, geminivirus infection could induce transcriptional silencing of transgenes containing cognate geminiviral sequences, which correlated with hypermethylation of these sequences at CG, CHG and CHH sites [51,69]. This process of virus-induced transgene silencing did not affect geminivirus symptom development or viral DNA accumulation [51]. Thus, while Rep-mediated replication rescues viral DNA from repressive methylation, Rep activity does not prevent *de novo* methylation and silencing of the target transgenes.

## 8. Plant Recovery from Geminiviral Infection and RdDM

Recovery from virus disease symptoms correlating with reduced viral titres has been observed for RNA and DNA viruses in certain host plants. For RNA viruses that spawn massive quantities of 21-nt and/or 22-nt siRNAs [2–5], the post-transcriptional RNA silencing pathway appears to mediate plant recovery [70]. For geminiviruses that spawn 24-nt siRNAs in addition to 21- and 22-nt siRNAs [58,71–73], both post-transcriptional and transcriptional silencing pathways have been implicated in recovery [72].

Evidence for a role of transcriptional silencing and cytosine methylation in the recovery process comes from several studies. Thus, plant recovery from infections with cassava mosaic begomoviruses correlated with increased accumulation of viral siRNAs of all the size-classes [73], suggesting that viral 24-nt siRNA may direct transcriptional silencing and thereby contribute to recovery. Recovery of pepper plants from begomovirus infection was associated with much low titres of both viral DNA and siRNAs in the youngest recovered leaves, compared to those in the severely infected old leaves. The levels of viral DNA methylation in all the contexts were elevated from ca. 10% in the old leaves to ca. 20% in the youngest leaves [72]. The inverse correlation between 5meC levels and 24-nt siRNA quantities in the respective leaves raises a question whether cytosine methylation is established through the action of viral 24-nt siRNAs. In another study, the abundance of begomovirus-derived siRNAs was also negatively correlated with plant recovery and positively correlated with viral titre [56].

The involvement of RdDM in plant recovery was deduced from the observation that contrary to wild-type plants, the mutant plants lacking AGO4 could not recover from infection with the BCTV mutant lacking L2. This correlated with lower 5meC levels of viral DNA in the disease-displaying *ago4* mutant plants (20%) than in the recovered wild type plants (80%) [52]. However, no difference in cytosine methylation of the wild type BCTV in *ago4* mutant plants *versus* wild-type plants was observed (18% in both cases). Since an AGO4-siRNA complex is a major effector of RdDM (Figure 1), it remains unclear which mechanism mediates cytosine methylation of geminiviral DNA and whether this mechanism requires viral 24-nt siRNAs.

Plant recovery from geminivirus infection can also be triggered by transient or stable expression of inverted-repeat transgenes that generate dsRNA cognate to the geminivirus intergenic region [74,75]. However, it is unclear whether dsRNA-derived 24-nt siRNAs or dsRNA itself contributed to the recovery and whether *de novo* methylation of the viral DNA plays a role in this process. Interestingly, the intergenic region of geminiviruses is a poor source of siRNAs (see below). Thus, targeting this naturally-protected region by artificial dsRNA could help the plant recover from the viral disease.

## 9. Genetic Requirements for the Biogenesis of Geminiviral siRNAs

Geminivirus-infected plants produce abundant virus-derived 21-, 22- and 24-nt siRNAs. As evaluated by deep sequencing, a sub-population of viral siRNAs can vary from ca. 1%–3% to 30%–50% of the total small RNA population in infected plants [76–78]. Thus, despite a tiny size of the geminivirus genome, the quantity of viral siRNAs in some virus-host systems (e.g., CaLCuV-infected *Arabidopsis* [78]) is comparable to a combined quantity of siRNAs and miRNAs expressed from the plant genome. It should be noted that the percentage of viral siRNAs in a total sRNA population is lower for those geminiviruses that are strictly limited to phloem tissues. Taking into account the dilution factor, geminivirus-infected phloem cells must produce massive amounts of viral siRNAs.

**Figure 3:**
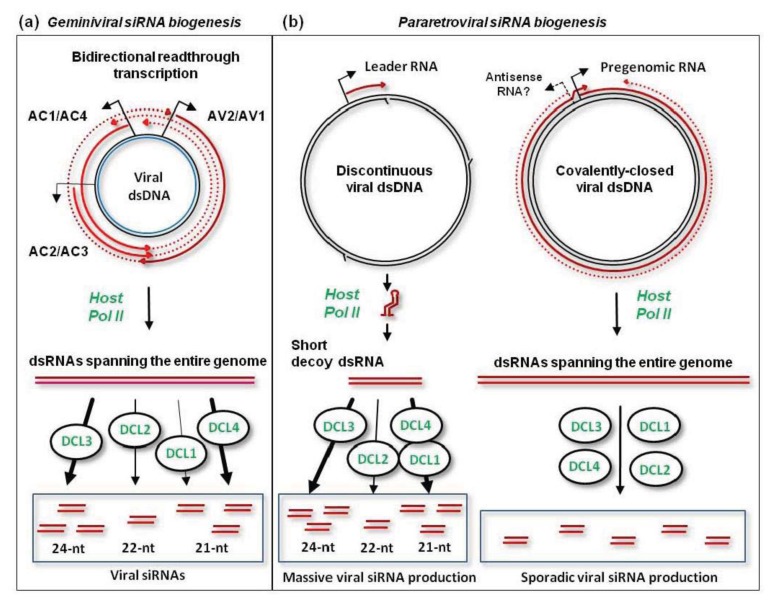
Models for the biogenesis of geminiviral and pararetorviral siRNAs. (**a**) The biogenesis of geminiviral siRNAs is initiated by bi-directional readthrough transcription beyond the poly(A) signals that normally terminate transcription of the viral leftward genes (in begomoviruses, AC1/Rep, AC4, AC2/TrAP and AC3) and the rightward genes (in begomoviruses, AV2 and AV1/CP). The resulting sense and antisense readthrough transcripts (dotted lines) anneal to the complementary viral mRNAs (solid lines with arrowheads) and to each other (in the intergenic region between the transcription start sites). This creates dsRNAs spanning the entire circular viral genome. Every DCL digests these dsRNAs into siRNAs of different sizes, with DCL3 (24-nt), DCL4 (21-nt) and DCL2 (22-nt) being favored (in that order); (**b**) Pol II transcribes both the discontinuous and the covalently-closed dsDNA forms of pararetrovial dsDNA. Abrupt termination of Pol II transcription at the unrepaired minus-strand DNA gap (Met-tRNA gap), results in production of aberrant 8S RNA lacking poly(A) tail (Leader RNA). This RNA forms a viroid-like secondary structure which can be converted by Pol II to dsRNA. The resulting dsRNA serves as a decoy to engage all the four DCLs in massive production of 21-, 22-, and 24-nt vsRNAs. Pol II-mediated transcription of the covalently-closed circular dsDNA generates pgRNA covering the entire genome as well as antisense transcript(s) (red dotted line). The 35S pgRNA promoter was reported to drive transcription not only in the forward but also in the reverse orientation [79] (indicated with bent lines with arrowheads). The pgRNA and antisense Pol II transcripts form low-abundance dsRNA spanning the entire virus genome. This dsRNA is diced by the four DCLs to generate viral 21, 22 and 24-nt siRNAs.

Although the hotspots of viral siRNA production are not equally distributed along the virus genome, unique (non-redundant) siRNAs of each size-class cover the entire circular viral DNA in both sense and antisense polarities, as demonstrated for CaLCuV DNA-A and DNA-B [78]. Based on genetic evidence combined with small RNA deep sequencing and blot hybridization, the biogenesis of CaLCuV siRNAs is mediated by all the four plant DCLs, but does not require RDR1, RDR2, RDR6, Pol IV, or Pol V [58,78]. The precursors of viral siRNAs are likely produced by Pol II-mediated bi-directional readthrough transcription of viral circular dsDNA far beyond the poly(A) signals (Figure 3A; further discussed in [4,78]). Such readthrough transcripts of sense and antisense polarities can potentially form dsRNA substrates for DCLs. Interestingly, the intergenic region harboring bidirectional promoter elements between the transcription start sites is a poor source of viral siRNAs [77,78]. This implies that the readthrough transcripts of each polarity are preferentially associated with more abundant viral mRNAs to form dsRNA. The resulting dsRNAs covering the leftward and rightward genes as well as less abundant dsRNAs covering the intergenic region are then processed by each of the four DCLs to generate 21-nt (DCL4 and DCL1), 22-nt (DCL2) and 24-nt (DCL3) siRNA duplexes [58,78] (Figure 3A). Both strands of these siRNA duplexes are then methylated at the 3′-terminal nucleotide’s hydroxyl by HEN1 [58] and presumably sorted by AGO proteins to form silencing complexes. By using CaLCuV as a vector for virus-induced gene silencing (VIGS) targeting a host gene, it was demonstrated that viral siRNA generated by each DCL has the ability to knock down target mRNA accumulation [58]. Furthermore, CaLCuV-VIGS targeting an enhancer region of 35S promoter-driven transgene could induce transcriptional silencing of the transgene in virus-infected plants [78]. Whether viral 24-nt siRNAs get associated with AGO4 to direct *de novo* methylation and transcriptional silencing remains to be investigated.

The most abundant viral 24-nt siRNAs, which can potentially direct *de novo* methylation of viral DNA, map to the coding regions of the geminivirus genome [77,78], where cytosine methylation may not affect viral transcription. Indeed, substantial methylation is found in the bodies of active *Arabidopsis* genes, *i.e.*, downstream of their promoters. In contrast, inactive, developmentally-regulated and tissue-specific genes tend to have high levels of cytosine methylation in the promoters [9]. As noted above the geminiviral bidirectional promoter region spawns low amounts of 24-nt siRNAs, which may not be sufficient for RdDM and transcriptional silencing.

Taken together, the biogenesis of geminiviral 24-nt siRNAs does not involve the core components of the RdDM pathway such as RDR2, Pol IV, or Pol V. Since these components are required for the biogenesis and function of endogenous 24-nt siRNAs, the RdDM pathway may not be effective in targeting viral dsDNA for cytosine methylation. This is consistent with the findings that circular viral dsDNA is not methylated. It remains to be investigated whether detectable methylation of viral heterogeneous linear dsDNA is established through RdDM. It is feasible that Pol II-mediated transcription of viral linear dsDNA may lead to targeting of the nascent transcript by viral 24-nt siRNA-AGO4 complexes, which would recruit DRM2. However, the resulting methylated DNA may not be able to recruit the Pol IV-RDR2 complex for dsRNA production and siRNA amplification, since this complex does not contribute substantially to production of viral siRNAs [58,78].

Notably, in the absence of three functional RDRs (RDR1, RDR2, RDR6), accumulation of CaLCuV siRNAs of all sizes was elevated, which correlated with increased accumulation of some viral transcripts [78]. Southern blot analysis revealed increased accumulation of viral circular ssDNA, but not circular dsDNA. This implies that plant RDR activities may repress both Pol II-mediated siRNA production via readthrough transcription and Rep-dependent production of circular ssDNA. Interestingly, a distinct plant siRNA-generating pathway has been implicated in recombination-dependent DNA repair [80]. It is tempting to speculate that viral siRNAs accumulating in the nucleus may facilitate viral DNA replication, e.g., by serving as primers for the host DNA polymerase.

## 10. Pararetrovirus Replication and Evasion of Transcriptional and Post-Transcriptional Silencing

Plants do not host retroviruses, but their genomes are populated by LTR retrotransposons whose transcriptional activity is repressed by RdDM. Only episomal pararetroviruses that do not obligatorily integrate into the host genome can replicate and spread in plants. The family *Caulimoviridae* comprises several genera of pararetroviruses with circular dsDNA gemomes of 7.4 to 8 kbp [81,82]. Like retroviruses, the pararetroviruses replicate via reverse transcription. The pararetroviral reverse transcriptase (RT) possesses RNA-dependent and DNA-dependent DNA polymerase activities and RNaseH activity, but lacks an integrase activity [81]. Nonetheless, some plant pararetroviruses have managed to integrate into the host genomes and form complex repetitive integration loci. Some of them, e.g., endogenous *Banana streak virus* and *Petunia vein clearing virus* (PVCV), can be released from the genome upon stress and cause disease [83,84].

**Figure 4:**
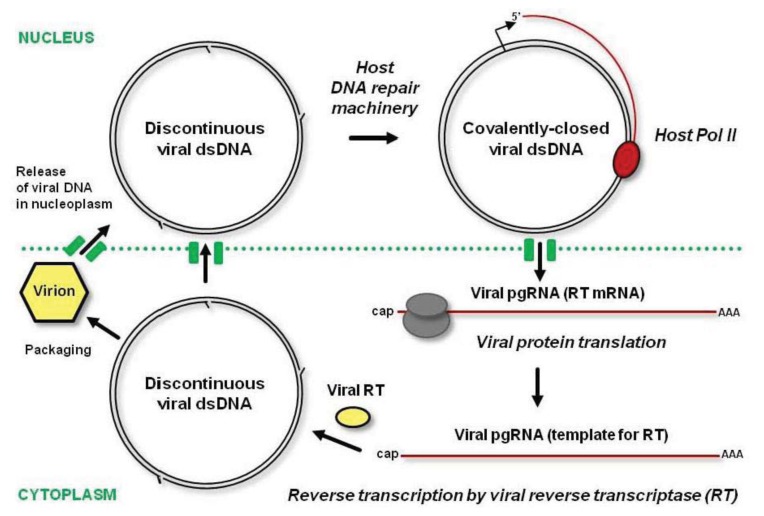
Model for pararetrovirus replication. Viral circular dsDNA from the virion (in yellow) is released into the nucleus. The gaps at both DNA strands are sealed by the host DNA repair machinery. The resulting covalently-closed dsDNA serves as a template for Pol II transcription generating viral pregenomic RNA (pgRNA). The capped and polyadenylated pgRNA is transported to the cytoplasm for translation of viral proteins including the reverse transcriptase (RT), and for subsequent reverse transcription catalyzed by RT. The resulting dsDNA with discontinuities at both strands can get packaged into a new virion or targeted to the nucleus for the next round of replication.

The genomic DNA of episomal pararetroviruses is encapsidated in icosahedral or bacilliform virions and transmitted from plant to plant by insect vectors [81]. Like in geminiviruses, a nuclear localization signal of pararetroviral coat protein promotes delivery of viral DNA into the nucleus. The virion-associated circular dsDNA has at least one gap (discontinuity) in each strand, the remnants from reverse transcription of viral pregenomic RNA (pgRNA) in the cytoplasm [85]. These gaps are sealed in the nucleus by the host DNA repair machinery and the resulting covalently-closed circular dsDNA gets associated with nucleosomes to form a viral minichromosome, the template for Pol II transcription (Figure 4). Pol II generates a capped and polyadenylated pgRNA that covers the entire virus genome and has a terminal redundancy, owing to the recognition of the poly(A) signal located at a short distance downstream of the transcription start site only on a second encounter. In some genera, Pol II transcription also generates a subgenomic RNA, the mRNA for P6/TAV protein. This multifunctional protein is involved in formation of dense inclusion bodies in the cytoplasm, translation reinitiation and suppression of plant defenses (see below).

The pgRNA harboring all the viral ORFs serves as an mRNA for polycistronic translation of viral proteins (including coat protein and RT) and as a template for reverse transcription. Following translation in the cytoplasm, the pgRNA is reverse transcribed by viral RT enzymatic activities with the help of coat protein. The resulting open-circular dsDNA with gaps at both strands can be delivered into the nucleus by coat protein, or get incorporated into a mature virion, which can re-infect the same nucleus or move out of the cell (Figure 4). As a result of multiple rounds of replication as well as cell-to-cell and long-distance movement of virions, the infected cells’ nuclei accumulate multiple copies of viral minichromosomes.

The cytoplasmic step of viral replication through pgRNA should effectively protect viral DNA from maintenance methylation and RdDM. However, covalently-closed circular dsDNA, which is transcribed in the nucleus, can potentially be methylated *de novo* by the RdDM machinery charged with viral 24-nt siRNAs. If this is the case, even inefficient transcription of viral minichromosomes with the repressive marks will generate pgRNA, and the next round of pgRNA translation and reverse transcription will produce unmethylated viral dsDNA.

Deep-sequencing analysis of small RNAs from *Arabidopsis* plants infected with *Cauliflower mosaic virus* (CaMV), a type member of genus *Caulimovirus*, has demonstrated that 21-, 22- and 24-nt viral siRNAs accumulate in massive quantities comparable to the entire complement of endogenous plant siRNA and miRNAs [86]. Moreover, massive production of all size-classes of viral siRNAs of both sense and antisense polarities is largely restricted to a 600 bp non-coding region of the CaMV genome, between the pgRNA transcription start site and the reverse transcription primer binding site. Other genomic sequences spawn much less abundant siRNAs of each size-class and polarity. Given that Pol II-mediated transcription of the CaMV genome generating pgRNA and P6 mRNA is mono-directional, the precursors of viral siRNAs covering the entire genome in both polarities are likely generated by antisense transcription driven by cryptic promoter(s) on viral DNA (Figure 3B). Alternatively, host RDR activities may convert viral RNAs into dsRNA. However, genetic evidence combined with siRNA deep sequencing and blot hybridization ruled out this hypothesis. Indeed, the biogenesis of viral siRNAs from both hot and cold regions does not require RDR1, RDR2, or RDR6 [58,86]. Furthermore, Pol V and Pol IV do not contribute to CaMV siRNA production. Hence, both sense and antisense strands of dsRNA precursors of viral siRNAs are likely generated by Pol II. The resulting dsRNAs are then processed by each of the four Dicers, which generate 21-nt (DCL1 and DCL4), 22-nt (DCL2) and 24-nt (DCL3) siRNAs [58,86]. DCL1, which normally generates plant miRNAs, produces a larger fraction of viral 21-nt siRNAs than DCL4 [58,86]. DCL4 is a primary dicer generating 21-nt siRNAs from RNA viruses [87] but its activity is inhibited by CaMV P6/TAV protein [88,89] (further discussed below).

The 600 bp non-coding region of CaMV genome generating the majority of viral siRNAs was proposed to produce a decoy dsRNA that would engage all the four DCLs and available AGOs in production and sorting of viral siRNAs [86] (Figure 3B). Such decoy strategy would protect other regions from silencing at both transcriptional and post-transcriptional levels. Indeed, the upstream pgRNA promoter elements and the downstream coding sequences spawn only small amounts of viral siRNAs that would have to compete with abundant, decoy dsRNA-derived siRNAs for AGOs to form silencing complexes. Consistent with the decoy model, immuno-precipitation with AGO-specific antibodies revealed that AGO1 is associated with 21-nt siRNAs from the non-coding region but not other regions of CaMV genome [86]. Surprisingly, only a tiny fraction of abundant 24-nt siRNAs from the non-coding region was associated with AGO4. AGO4 complexes in the nucleus are likely saturated with endogenous 24-nt siRNAs and only a small pool of free AGO4 is available. If the non-coding region becomes *de novo* methylated through the action of detectable silencing complexes, transcriptional activity of the upstream promoter will not be affected. At the post-transcriptional level, the 600 nt non-coding leader sequence of pgRNA folds into a stable secondary structure bypassed by ribosomes to initiate translation [90–92], which may not be accessible for 21-nt siRNA-AGO1 complexes. Taken together, the decoy strategy evolved by CaMV [86] and possibly other pararetroviruses with a similar configuration of the non-coding region elements and structures [93] would help the virus evade silencing at both transcriptional and post-transcriptional levels.

Like in the case of geminiviruses, pararetrovirus infection can induce silencing of transgenes sharing homology with the virus. In CaMV-infected plants, the transgenes driven by the CaMV 35S pgRNA promoter were silenced at the transcriptional levels, whereas those with the CaMV 3′UTR sequences at the post-transcriptional level [94,95]. Notably, CaMV replication and viral transcript accumulation were not affected by ongoing silencing of the transgenes [94]. Thus, CaMV can indeed evade both transcriptional and posttranscriptional silencing as argued above. It remains to be investigated if silencing of homologous transgenes is directed by viral siRNAs.

Some host plants can recover from pararetrovirus disease symptoms, but abundant viral dsDNA can still persist in the recovered tissues. The recovery of kohlrabi plants from CaMV infection was preceded by overaccumulation of covalently-closed viral dsDNA in the nucleus, followed by arrest of reverse transcription [96]. Interestingly, overall transcription of viral dsDNA in the nucleus (evaluated by a “nuclear run-on” method) did not change after the transition to recovery, but accumulation of polyadenylated viral transcripts was strongly reduced. This implicates post-transcriptional silencing in the recovery process. Notably, covalently-closed viral dsDNA was not found to be methylated before or after recovery [96]. The mechanisms underlying the overaccumulation of viral minichromosomes before recovery and the posttranscriptional degradation of viral RNAs remain to be further investigated.

Endogenous pararetroviruses integrated in the host genomes are likely repressed by cytosine methylation and histone modifications. These repressive marks can potentially be established *de novo* by RdDM and efficiently maintained following plant DNA replication. The integrated copies of PVCV in the petunia genome were found to be associated with repressive H3K9me2 marks [84]. In this case, accumulation of 21–24 nt viral siRNAs was barely detectable, and only disease induction could boost viral siRNA production. Hence, the released episomal virus spawns much more abundant siRNAs than the integrated copies. Deep-sequencing of siRNAs combined with cytosine methylation analysis should clarify whether the infectious copies of integrated pararetroviral DNA are densely methylated and whether cytosine methylation is established and maintained by RdDM. In the case of an endogenous tomato pararetrovirus, which cannot be released as episomal virus, the integrated viral sequences were found to be methylated at CHG and CHH sites and virus-derived 21–24 nt siRNAs accumulated at detectable levels [97].

## 11. Suppression of Plant Defenses by Pararetroviral Proteins

CaMV P6/TAV protein has been implicated in suppression of the plant defenses based on RNA silencing [88,89,98] and innate immunity [8,99]. Since this protein has no homologs in several genera of *Caulimoviridae* [81], plant pararetroviruses have to rely on other strategies to suppress or evade plant defenses. The tungrovirus *Rice tungro bacilliform virus* possesses a P4 gene of unknown function, which is missing in closely related badnaviruses. Like CaMV P6 gene, the P4 gene is located downstream of the RT gene and expressed from a separate mRNA [81]. These similarities suggest that P4 may have been acquired by a badnavirus to cope with plant defenses in a new host.

The mechanism of silencing suppression by CaMV P6/TAV has been extensively investigated [88,89,98]. According to the current model, P6 interferes with amplification of secondary siRNAs by blocking DCL4-mediated processing of RDR6-dependent dsRNAs. Curiously, nuclear import of P6 was required for P6-mediated suppression of endogenous tasiRNA biogenesis, which presumably occurs in the cytoplasm, and for P6 interaction with DOUBLE-STRANDED RNA BINDING 4 (DRB4), a partner of DCL4 [88]. It remains to be demonstrated if these activities of CaMV P6 are also required for suppression of antiviral silencing. Indirect evidence supporting this hypothesis is that only a fraction of CaMV 21-nt siRNAs is produced by DCL4 and the biogenesis of the DCL4-dependent fraction of viral 21-nt siRNAs does not require RDR6 activity [58,86]. However, presumptive RDR6-dependent precursors of viral secondary siRNAs, which should be stabilized by the P6 action, could not be detected in CaMV-infected plants. In contrast the RDR6-dependent dsRNA precursors of plant tasiRNAs are readily detectable in both CaMV-infected and P6 transgenic plants [19,20,58,89]. Thus, CaMV infection or P6 expression does not interfere with RDR6 activity, but viral mRNAs (and their degradation products) appear to be poor substrates for RDR6. Similar findings have been reported for the geminivirus CaLCuV [78]. Thus, DNA viruses have evolved to protect their mRNAs from RDR activity that would amplify and spread antiviral siRNAs. Likewise, most of the plant genes controlled by miRNAs do not spawn RDR6-dependent secondary siRNAs.

The caulimovirus P6/TAV is a multifunctional protein harboring the domains implicated in interactions with ribosomal proteins and translation initiation factors, in binding RNA (single and double-stranded), in formation of inclusion bodies, and in hypersensitive immune responses [100–102]. The domain responsible for suppression of RNA silencing has not been identified yet.

It has been hypothesized that P6/TAV and suppressor proteins of other plant viruses can interfere with the innate immune responses, which restrict growth of non-viral pathogens [8]. In resistant hosts, CaMV P6 triggers hypersensitive responses and its avirulence domain recognized by the immune system has been mapped. Notably, this P6 domain is also required for CaMV virulence in susceptible hosts [101]. By analogy with effector proteins of non-viral pathogens, a primary function for P6 is to suppress basal immune responses. In resistant hosts, P6 effector activity is recognized by the immune receptors of the NB-LRR family, which triggers hypersensitive response and programmed cell death restricting viral infection [8]. This hypothesis is supported by the finding that CaMV P6 expression in transgenic plants promotes growth of a bacterial pathogen [99]. The mechanism of P6 interference with the immune responses remains to be investigated.

## 12. Concluding Remarks

Unlike animals, land plants do not host “true” dsDNA viruses whose replication mechanisms generate dsDNA genome copies without a ssDNA or RNA intermediate, or “true” retroviruses with a provirus stage of replication that involves viral DNA integration in the host genome. This exclusion is likely because the land plants have evolved the mechanisms of siRNA-directed *de novo* methylation of all cytosines (RdDM) and maintenance methylation at both CG and non-CG sites. These mechanisms establish and maintain cytosine methylation in all sequence contexts of the plant genome and thereby effectively repress unwanted transcription in the nucleus. This repressive methylation system is evaded by ssDNA viruses which can resurrect their dsDNA forms from cytosine methylation by Rep-dependent replication generating unmethylated ssDNA. Likewise, pararetroviruses that omit a host genome integration step can thereby evade the transcriptional silencing reinforced by a concert action of maintenance methylation and RdDM-dependent amplification of siRNAs. Furthermore, episomal pararetroviruses can evade repressive methylation by constant delivery of multiple unmethylated copies of circular dsDNA to the nucleus from the cytoplasm where pgRNA is reverse transcribed. Having these replication strategies, pararetroviruses and ssDNA viruses have not been under a strong pressure in land plants to evolve suppressors of cytosine methylation.
